# Use of Nutraceuticals in Angiogenesis-Dependent Disorders

**DOI:** 10.3390/molecules23102676

**Published:** 2018-10-18

**Authors:** Lucia Morbidelli, Erika Terzuoli, Sandra Donnini

**Affiliations:** Department of Life Sciences, University of Siena, Via A. Moro 2, 53100 Siena, Italy; terzuoli8@unisi.it (E.T.); sandra.donnini@unisi.it (S.D.)

**Keywords:** angiogenesis, nutraceutical, cancer, endothelial dysfunction, inflammation, oxidative stress

## Abstract

The term of angiogenesis refers to the growth of new vessels from pre-existing capillaries. The phenomenon is necessary for physiological growth, repair and functioning of our organs. When occurring in a not regulated manner, it concurs to pathological conditions as tumors, eye diseases, chronic degenerative disorders. On the contrary insufficient neovascularization or endothelial disfunction accompanies ischemic and metabolic disorders. In both the cases an inflammatory and oxidative condition exists in supporting angiogenesis deregulation and endothelial dysfunction. The use of nutraceuticals with antioxidant and anti-inflammatory activities can be a therapeutic option to maintain an adequate vascularization and endothelial cell proper functioning or to blunt aberrant angiogenesis. A revision of the updated literature reports on nutraceuticals to guide endothelial cell wellness and to restore physiological tissue vascularization is the objective of this paper. The critical aspects as well as lacking data for human use will be explored from a pharmacological perspective.

## 1. Angiogenesis Definition and Mechanisms

Angiogenesis, mainly induced by hypoxia in growing, remodelling or ischemic tissues, is defined as the growth of new capillaries from the pre-existing blood vessels. The neovascular growth involves the proliferation, migration, and differentiation of vascular endothelial cells (ECs) under the stimulation by specific angiogenic factors and following defined biochemical pathways and molecular mechanisms [1,2]. Angiogenesis begins with proangiogenic growth factors binding to their receptors in ECs, which in turn activate the same ECs, inducing the release of matrix metalloproteinases (MMPs). These proteases then degrade the capillary basement membrane and allow the activated ECs to migrate and proliferate outside of the original blood vessel. Then, with the participation of adhesion molecules (e.g., integrin αvβ3 and αvβ5) and MMPs, the sprouting vessels are extended and remodeled. ECs are then connected to each other to form a tube or a loop, and finally, pericytes are incorporated to stabilize the newly formed capillaries [3]. 

Numerous are the factors found to promote or inhibit angiogenesis. All these molecules with opposite effects control angiogenesis in a balanced manner and a shift in this balance can lead to pro- or anti-angiogenic effects. The angio-modulatory factors are released by inflammatory and stromal cells, neoplastic and other diseased cells, in response to inflammation, hypoxia, and other pathophysiological conditions, [4,5,6].

Vascular endothelial growth factor (VEGF) is reported as the main growth factor with high specificity for the vascular endothelium. The expression of its receptors (VEGFR) has been considered a critical event in endothelial cell activation. Other growth factors contribute to the vascular development and promote angiogenesis, such as fibroblast growth factors (FGF-1 and FGF-2), platelet-derived growth factor (PDGF), transforming growth factor-β (TGF-β), angiopoietins (Ang-1 and Ang-2) and tyrosine kinase receptors tie-2, expressed in vascular and stromal cells. When inflammation is the trigger of angiogenesis outcome, other mediators are involved as cytokines, prostaglandins [7,8] and nitric oxide (NO) [9,10] which mediate and amplify the angiogenic activity of growth factors [11,12,13]. Other types of chemokines on the contrary negatively control angiogenesis, together with known antiangiogenic factors, such as angiostatin, endostatin, thrombospondins, pigment epithelium-derived factor [5,6].

## 2. Angiogenesis Related Diseases

**Figure 1:**
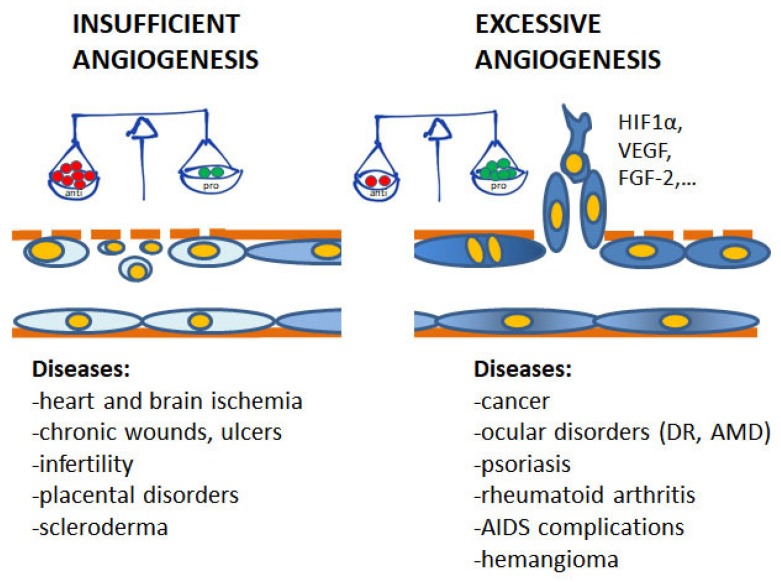
Contribution of insufficient and excessive angiogenesis to different pathological disorders. At the base of the angiogenesis outcome is the unbalance between pro- and antiangiogenic molecules, induced by conditions of hypoxia, metabolic disorders, inflammation and oxidative stress. (DR, diabetic retinopathy; AMD, age-related macular degeneration).

Angiogenesis plays pivotal physiological functions in placenta formation, embryonic development, wound repair, and tissue remodeling, regeneration and engineering [1]. During embryo growth, angiogenesis (occurring after vasculogenesis) is essential for the proper development of a functional circulatory system, delivering oxygen and nutrients to every cell of the body and allowing waste product removal [6]. In contrast, in the adult, only few physiological processes are angiogenesis-related as the reproductive cycle, wound healing and bone repair. In all these events, angiogenesis occurs transiently and is finely regulated by the balance between pro- and antiangiogenic factors. Nevertheless, when dysregulated, neovascularization contributes to several oncogenic, ischemic, inflammatory and infectious diseases [5,6]. The list of angiogenesis-dependent disorders in quite long and includes apparently unrelated disorders, including proliferative retinopathies, rheumatoid arthritis, psoriasis, endometriosis, and the most of cancers. As depicted in Figure 1, in these angiogenesis-dependent diseases, an imbalance of pro-angiogenic regulatory factors over anti-angiogenic ones has been documented, leading to the so-called angiogenic switch [1]. On the contrary, there are pathologies such as ischemic diseases which result in a deficient blood supply due to vessel degeneration. In these cases, neovascular restoration is needed. 

In addition, we have to consider a subtle pathophysiological condition which is referred as endothelial dysfunction, meaning the failure of endothelial cells to maintain vascular homeostasis. Endothelial dysfunction is a systemic pathology that modify endothelial cell phenotype, resulting in reduced vasodilation, proinflammatory and prothrombic features. Endothelial dysfunction is induced by various conditions including turbulent blood flow, shear stress, hypoxia, ageing, hyperglycemia, hypercholesterolemia and hypertension [14,15]. In particular, endothelial dysfunction in diabetes is an early event leading to microvascular and macrovascular complications. The microvascular complications in diabetes comprise enduring alterations in capillaries including diabetic retinopathy, nephropathy and neuropathy, accompanied by an impaired healing of wounds that become chronic. The macrovascular complications are related to diseases of large blood vessels, responsible for cardiovascular, peripheral vascular and cerebrovascular disorders, for which diabetic patients are at high risk.

In principle, pharmacological interventions on angiogenesis-dependent diseases can include compounds or strategies able to negatively control the angiogenic balance (anti-angiogenic therapy) or, on the contrary, to stimulate angiogenesis in the case of diseases with insufficient blood supply or requiring endothelization of compromised vessels (pro-angiogenic therapy). These opportunities justify the great attention on angiogenesis, maintained for the last decades by pharmaceutical companies and research groups devoted to develop compounds or strategies able to affect this process [16,17,18].

At present, both anti- and pro-angiogenic strategies have been developed for human diseases. While inhibition of angiogenesis has been shown to have success in disorders such as cancer, hemangioma, ophthalmic diseases, arthritis and psoriasis [19], few treatment protocols have reached the clinic with the aim to stimulate angiogenesis in ischemia-associated diseases [20,21]. Nevertheless, preventive or curative treatments to maintain endothelial integrity and functionality are factual medical needs. 

Beside the success of antiangiogenic drugs, relevant problems remain to be solved as resistance occurrence and relevant side effects (i.e., hypertension development following bevacizumab systemic treatment). The research is indeed active in exploring molecules able to affect or promote angiogenesis. Our attention has been focused on nutraceuticals since they promise to be a good source of therapeutic and preventing strategies in most of the diseases associated with aberrant or not functioning vessels.

## 3. Nutraceuticals

Even if the term is highly debated, the classical definition of nutraceutical is a foodstuff (such as a fortified food or dietary supplement) that provides health benefits in addition to its basic nutritional value. It is nowadays consolidated the concept that the diet components affect our health both predisposing to diseases (obesity, atherosclerosis, hypertension, diabetes, cancer, among the others, induced by foods particularly rich in saturated fats and carbohydrates), and preventing them (see the beneficial effects of Mediterranean diet on the same above diseases). Thus, the finding of active principles (and their proper formulation) designed to prevent or cure a certain disorder is an important medical opportunity. Moreover, since most of the nutraceuticals derive from consolidated ethnical and popular uses, the research costs for development are reduced, thus allowing to propose this type of medical care to a large number of people and to disadvantageous populations.

Nutraceutical compounds are of different nature, represented by ω-3 fatty acids, aminoacids and proteins, vitamins and minerals, fibers and polyphenols and other secondary metabolites [22,23]. Sources of nutraceuticals are plants, animals and microorganisms (bacteria, yeasts, fungi) and there are cases in which the active moiety derives from metabolism due to environmental microorganisms or endogenous microbiota. 

Polyphenols are natural compounds which consist of one or more than one benzene rings bearing one or several hydroxy functions. They are a large family of different substances derived from the metabolism of shikimic acid and/or polyacetate, common in all vascular plants [24]. Natural polyphenols are found in high quantities (up to 200–300 mg polyphenols per 100 g fresh weight) in fruits, vegetables and cereals and products prepared from these foods. Chocolate also contributes to the daily polyphenol intake [25,26]. Plants produce polyphenols as secondary metabolites in order to protect against ultraviolet radiation or aggression by pathogens [27]. However, in foods, polyphenols can cause the flavor, odor, color, bitterness, astringency and stability to oxidation.

It is well known that the long duration consumption of plants rich in polyphenols reduces the risk of developing cancers, cardiovascular pathologies, diabetes, osteoporosis and neurodegenerative disorders [28]. Due to the possible beneficial effects on human health, polyphenols and other phenolic food components are a hot topic for the scientific community [29,30]. 

Polyphenols are the most studied nutraceuticals so far and many beneficial effects on various diseases have been reported in the literature, along with the problems and limits related to their use as drugs [30]. We refer to recent reviews for their anti-cancer effects, as they are able to modulate multiple coding and non-coding genes, ultimately targeting the main hallmarks of cancer [31,32,33,34,35]. 

For most of them, antiangiogenic properties have been reported and discussed [36,37], along with reversibility of endothelial dysfunction in diabetes and other cardiovascular disorders [38]. 

In this review we will focus on recent findings on less known polyphenols and other nutraceuticals, considering their anti- and proangiogenic applications. Both recent literature and clinical trials (2016–2018) have been taken into consideration.

Currently, there are 231 clinical trials with nutraceuticals. The majority are focused on nutraceutical efficacy on cardiovascular diseases, including hypercholesterolemia, hyperlipidemia, diabetes, and hypertension, but there are also studies on diabetic retinopathy, dementia, brain injury, pain and cancer. Thirty-four trials are recruiting and 130 are completed, and among these, four trials have been conducted to assess the efficacy of nutraceuticals on endothelial (dys)-function (NCT02969070; NCT00654459; NCT00296595; NCT01085019). However, although the last three trials are completed, the results have not been posted. A representative list of clinical trials on nutraceuticals effect in diseases characterized by dysfunctional angiogenesis is reported in Table 1.

### 3.1. Nutraceutical Antiangiogenic Strategies

This section includes reports on newly described nutraceuticals with antiangiogenic effect proposed both as prophylactic and curative strategies in different pathologies often accompanied by inflammation and an oxidative stress environment. A partial list of food and their major nutraceutical components shown to have antiangiogenic properties is reported in Table 2. 

For average concentration of each active molecule and specific antiangiogenic mechanism in experimental models, see details in [39]. 

#### 3.1.1. Chemopreventive and Antitumor Approaches

Bathia and coworkers report the antitumor effect of lycopene-enriched tomato extract (LycT) by affecting hypoxia-induced factor (HIF)-1α, VEGF, CD31, MMP-2 and MMP-9, in the initial steps of liver carcinoma chemically induced in mice, providing evidence that prophylactic dietary supplementation with LycT may counteract HCC progression and/or protect against tumor onset [40].

Another interesting example of chemopreventive and therapeutic efficacy of a nutraceutical approach is represented by blueberry supplementation in animal carcinogenesis model which was reported to inhibit the development and progression of squamous cell carcinomas by abrogating TGF-β and PI-3K/Akt pathways and downregulating MMPs and VEGF. Blueberry extract also inhibited migration and tube formation of cultured ECs [41].

The Mediterranian diet is a well-established alimentary style with protective efficacy against different metabolic disorders. Among the diet components, extra virgin olive oil (EVOO) is an interesting source of active principles [42]. From a chemical point of view, 98–99% of the total weight of EVOO is represented by fatty acids, especially monounsaturated fatty acids such as oleic acid. Tocopherols, polyphenols and other minor constituents represent the remaining 1–2%. Recently, the anticarcinogenic effects of olive oil phenolic alcohols and their secoiridoid derivatives have been established by the use of different experimental models, demonstrating their capacity to inhibit proliferation and invasion of cancer cells, induce apoptosis, block tumor angiogenesis and regulate inflammatory response. In some cases the molecular mechanisms were not directly associated to their anti-oxidant effects. 

We have contributed to demonstrate that dihydroxyphenylethanol or hydroxytyrosol (HT), a product from olive oil, blocks microsomal prostaglandin-E synthase-1 and HIF-1α dependent VEGF expression, thus reducing tumor angiogenesis [43], and promotes epidermal growth factor receptor (EGFR) degradation in colon cancer tumor cells, thus sensitizing them to anticancer drugs [44,45].

Oleuropein (OL), the most abundant phenolic compound in olives, was demonstrated to inhibit progression of melanoma in mice exposed high-fat-diet (HFD)-induced obesity. OL suppressed HFD-induced tumor growth by reducing the expression of angiogenesis (CD31, VE-cadherin, VEGF-A, and VEGFR2), lymphangiogenesis (LYVE-1, VEGF-C, VEGF-D, and VEGFR3), and hypoxia markers (HIF-1α and GLUT-1). Additionally, OL directly inhibited in vitro tube formation of HUVECs and lymphatic ECs [46]. 

Other plant derivatives recently described with antitumor and antiangiogenic efficacy in cellular and animal models were hydroxybenzoic acids, hydroxycinnamic acids and flavone groups from the aqueous extracts of *Basella alba* and *B. rubra* stems [47], methanol extract of wheat grass [48], bioactive compounds derived from *Allium* vegetables such as diallyl trisulfide (DATS) [49], and capsaicin [50], which act on multiple molecular targets associated with cancer progression and metastasis.

Another interesting nutraceutical approach is the possibility to access to antiangiogenic proteins present in milk. Milk contains at least three proteins with modulatory effect on angiogenesis [51]. The antiangiogenic activity of milk lactoferrin, known from 1997, has been confirmed recently in experimental human colon cancer models, consistent with a significant downregulation of VEGFR2, VEGF-A, pPI-3K, pAkt, and pERK1/2 proteins [52].

This limited list of examples comes from epidemiologic studies which linked consumption of certain vegetables or foods to decreased incidence of cancer and pave the way for the development of defined products with high significance in the pharmaceutical and nutraceutical industries.

#### 3.1.2. Nutraceuticals and Ocular Disorders

Various eye diseases are characterized by progressive evolution accompanied by inflammation and oxidative stress. Thus, they are the ideal target for antioxidant and anti-inflammatory nutraceuticals.

Age-related macular degeneration (AMD) is a progressive eye disease typical of the elderly, affecting the macula, the central region of the retina. In AMD patients, degeneration affects firstly the retinal pigment epithelial cells and then the photoreceptors, leading to alteration or partial loss of central vision and blindness. Two forms of AMD can be identified: the more frequent atrophic-dry AMD and the less frequent neovascular-wet AMD characterized by choroidal neovascularization (CNV). The pathogenesis of AMD is complex and multifactorial. The recognized risk factors include: genetic predisposition, environmental determinants (i.e., intensive light exposure) and life style (i.e., smoking). The development of AMD is typically accompanied by the molecular processes of lipofuscinogenesis, drusogenesis and inflammation, while in wet AMD angiogenesis unbalance is typically found.

Diabetic retinopathy (DR) is broadly recognized as a microvascular complication of diabetes. Clinically, DR can be classified into non-proliferative DR (NPDR) and proliferative DR (PDR). NPDR is characterized by the occurrence of microaneurysms and small hemorrhages. Severe NPDR shows increased retinal microvascular damage as evidenced by cotton wool spots, venous beading and loops and abnormalities in retinal capillaries. Reduced perfusion and degeneration of the retinal capillaries bring to a status of hypoxia and activation of HIF-1α. Indeed, if left untreated, PDR can develop with anomalous retinal neovascularization, retinal edema, vitreous hemorrhage and tractional retinal detachment, till irreversible blindness in working age. As for AMD, oxidative stress and inflammation are recognized events occurring in DR. Beside glycemia control, it is important to preserve microvascular functioning in the first stage and then inhibit neovascularization in PDR.

Since VEGF is a predominant proangiogenic factor in choroidal and retinal neovascular growth, wet AMD and PDR are treated with intravitreous injections of anti-VEGF agents, but due to side effects and costs, effective and safe alternatives are needed. Nutraceuticals can be a chemopreventive or complementary approach.

Attention has been devoted to polyphenols (resveratrol, curcumin as example) [53] and ω-3 polyunsaturated fatty acids. In particular, dietary long-chain ω-3 polyunsaturated fatty acids (LCω-3PUFAs) and lutein have been reported to protect against AMD. Yanai et al. [54] demonstrated lower levels of various inflammatory modulators in the retina or choroid in mice fed with LCω-3PUFAs or lutein, without additive effects. On the contrary, the generation of reactive oxygen species (ROS) in experimental chorioretinal lesions, as well as the expression of NADPH oxidase 4 in the retina of mice were attenuated by LCω-3PUFAs and lutein in a synergistic manner. Similarly, curcumin decreases ROS generation and TNF-α release in human retinal endothelial cells and epithelial pigmented cells exposed to oxidative stress, and protects pericytes from high glucose induced damage [55]. Of note, curcumin supplementation is currently under clinical evaluation in DR patients (NCT02984813 and NCT01646047 clinical trials) (Table 1).

These results thus show that supplementary oral dietary intake of LCω-3PUFAs, lutein and curcumin attenuates ocular diseases, including CNV and diabetic retinopathy. The protective effects seem to be additive and associated to downregulation of inflammatory mediators and ROS [50,51]. 

### 3.2. Nutraceuticals and Pro-Endothelium Applications

In the following section we will report on some nutraceutical interventions in various angiogenesis disorders where endothelial integrity needs to be recovered or physiological angiogenesis promoted. A partial list of foods and their major nutraceutical components demonstrating to have proangiogenic and endothelial protective activities is reported in Table 3.

For average concentration of each active molecule and specific endothelium protective mechanisms, see details in [56,57].

#### 3.2.1. Interventions for Endothelial Dysfunction

In the last few decades, numerous epidemiological studies, as well as interventional trials, confirmed cardioprotective properties of the Mediterranean diet [58]. In this context, EVOO, the most representative component of this diet, seems to be relevant in lowering the incidence of cardiovascular events, including myocardial infarction and stroke. Oleic acid, tocopherols and polyphenols present in EVOO may potentially contribute to ‘health maintenance’ [59]. In addition, the protective effect of nuts and apple bioactives on vascular oxidative stress and endothelial functioning has also been recently confirmed [58,60].

Vitamins should be included in this dissertation. Recently, epidemiological studies have identified an inverse relation between cardiovascular health and folic acid intake and plasma folate levels. In addition, experimental studies were conducted to elucidate the mechanism(s) through which folic acid improves vascular endothelial function. These studies document that folic acid and its active metabolite 5-methyltetrahydrofolate increase NO bioavailability by triggering endothelial NO synthase (NOS) coupling and improving NO production, as well as by directly scavenging ROS [61]. By these mechanisms, folic acid may protect or improve endothelial function, thereby preventing or reversing the progression of cardiovascular disorders in patients with overt disease or at elevated risk. These findings encouraged a number of clinical studies on folic acid supplementation to potentially reverse endothelial dysfunction in patients with cardiovascular pathologies, which are ongoing with positive outcomes [62].

The same evidence seems not so clear for vitamin D, C and E. In normotensive subjects, reduced levels of 25-hydroxyvitamin D (25[OH]D) have been associated with an increased risk of hypertension, while deficiency of vitamin D has been correlated with endothelial dysfunction. Nevertheless, the effect of vitamin D supplementation on endothelial dysfunction in normotensive subjects has not been evaluated rigorously. Results from a recent clinical trial documented however no improvement in endothelial function after administration of vitamin D in overweight/obese normotensive peoples [63]. Vitamin C and E demonstrated negative results on clinical trials, and their preventive efficacy in people at cardiovascular risk is still debated in the literature [64].

#### 3.2.2. Nutraceutical Approaches for Diabetes Mellitus

Diabetes mellitus (DM) is characterized by chronic damage to endothelium, especially of microvessels, due to hyperglycemia. The damage to endothelium causes endothelial dysfunction, thus predisposing patients to a series of vascular and neurovascular pathologies, in which the vascular wall is characterized by a persistent condition of inflammation and oxidative stress. One of the consequences is impairment of wound healing and tissue regeneration [65].

Endothelial progenitor cells (EPCs), present in bone marrow and peripheral blood, as well as other mesenchymal stem cells, are involved both in the repair of damaged vessels and in neovascularization. Their activity should be improved in DM patients with vascular complications since the advanced glycation end products (AGEs) generated in DM subjects affect the number and the function of EPCs. Thus, the availability of compounds with anti-oxidant, anti-inflammatory and pro-survival or proangiogenic properties is crucial to limit vascular complications in DM patients.

Lycopene (Lyc) is an identified natural antioxidant that protects EPCs from damage due to microenvironmental AGEs. However, the underlying mechanism remains to be defined. Recently, Zeng and coworkers demonstrated that Lyc improved EPC proliferation and reduced cytotoxicity due to AGEs in type 2 diabetes mellitus rats. In particular, activation of cell cycle, reduced apoptosis and decreased autophagic reaction including ROS and mitochondrial membrane potential were found in Lyc exposed EPCs [66]. By promoting EPCs survival and protecting EPCs from apoptosis and oxidative autophagy induced by AGEs, Lyc supports the number and function of EPCs, suggesting to be a new potential therapeutic option for DM vascular complications.

Another interesting approach is represented by polyunsaturated fatty acids (PUFAs), specifically the ω-3, which are essential for growth and development, as during pregnancy. Mathew and coworkers treated placenta-derived mesenchymal stromal cells with ω-3 fatty acids docosahexaenoic acid (DHA) and eicosapentaenoic acid (EPA), testing their angiogenic potential. They reported an upregulation of both FGF-2 and VEGF-A and enhanced in vitro angiogenesis with DHA:EPA, also in coculture experiments with mature ECs. Their finding supports the supplementation with low doses of PUFAs in enhancing the angiogenic potential of placenta derived mesenchymal stromal cells [67], making them appealing as cellular therapy to favor wound healing in chronic metabolic disorders characterized by poor recovery of tissue integrity [65]. 

#### 3.2.3. Nutraceutical Control of Placenta Development and Preeclampsia

Inflammatory, angiogenic and apoptotic processes influence the normal vascular development of placenta. Altered placenta structure and function can be the cause for fetal growth retardation, blood clotting, preeclampsia and hypertension. Indeed, preeclampsia is a disorder of pregnancy, associated with altered angiogenesis and an inflammatory condition. Currently, it is well established that a critical feature of preeclampsia is endothelial dysfunction [68].

Asymmetric dimethylarginine (ADMA), a methylated metabolite of the amino acid l-arginine is an endogenous inhibitor of NOS. ADMA high levels contribute to endothelial dysfunction and inhibit angiogenesis and arteriogenesis, events essential during pregnancy and deficient in preeclampsia. Indeed, pregnant women with high ADMA concentrations at the early phase, later develop preeclampsia [69]. The randomized placebo-controlled clinical trial NCT02772887 has been designed as supplementation of l-citrulline in pregnant women with type 2 diabetes (Table 1), to evaluate whether l-citrulline could normalize plasma l-arginine/ADMA ratio, decrease maternal blood pressure and rise the levels of placental growth factor (PLGF), a marker of placenta functioning and angiogenesis.

For other nutraceuticals the experimental approach is on animal models. The effect of the micronutrients vitamin B12 and folate, and ω-3 fatty acid supplementation (separate, as well as combined) on genes regulating angiogenesis and inflammation and DNA methylation degree has been evaluated in a rat model of pregnancy induced hypertension [70,71]. The combined use of folic acid, vitamin B12 and ω-3 fatty acids improved IL-10 and VEGF placental levels and decreased TNF-α levels in offspring tissues, indicating that this nutraceutical mix can be beneficial in preeclampsia management. However, only appropriately designed randomized prospective clinical studies will ultimately validate these findings.

#### 3.2.4. Vascular Ageing and Cerebrovascular Interventions

Ageing is characterized by a decline in the functions of all systems, including the cardiovascular one. Age-related endothelial dysfunction is caused by compromised autophagy and oxidative stress. ROS reduce the bioavailability of NO, whose downregulation has been associated with compromised vasodilatory response, hypertension and arteriosclerosis. High levels of free radicals and low bioavailability of NO start a feedback loop of further oxidative stress, biochemical alterations, organelle damage and endothelial dysfunction. In addition, a greater susceptibility to apoptosis and senescence may contribute to proliferative and functional impairment of EPCs. Indeed, vascular ageing is associated with alteration in the structure and function of vessel wall and with impairment in angiogenesis and tissue repair. Inflammageing, the chronic low-grade inflammatory condition typical of elderly people, worsens vascular pathology and induces atherosclerosis, the major disorder at cardiovascular and neurovascular level. Nutraceuticals may represent a potential treatment in the prevention or delay of vascular ageing and Mediterranean diet seems a good strategy to preserve vascular wellness [72].

Treatment with resveratrol, mainly contained in grapes and berries, has been reported to stimulate autophagy and to restore oxidative balance in cultured endothelium [73], suggesting a therapeutic potential to recover endothelial function in the elderly. 

Literature consistently reports that long-chain ω-3 polyunsaturated fatty acids (LCω-3PUFA) may improve brain functions by acting on cerebrovascular ECs to facilitate vasodilatation and perfusion, thus reducing senescence and neurodegeneration [74].

In a recent report, forty randomized controlled clinical trials examining the effect of LCω-3PUFA use on either endothelial vasodilation function or cognition, were selected and analyzed [75]. Compared to placebo, an improvement of flow-mediated dilatation and cognitive function was found in subjects undergoing LCω-3PUFA supplementation [75]. This report however is in contrast with previous metanalysis which failed to demonstrate that ω-3 fatty acids, B vitamins, and vitamin E supplementation were able to affect cognition in non-demented middle-aged and older adults [76] and the debate is still open.

Dietary intake of ω-3 polyunsaturated fatty acids (ω-3PUFAs) has been shown to significantly prevent ischemic brain injury. The therapeutic value of ω-3PUFA administration has been characterized by Pu and colleagues in post-stroke animals, especially evaluating its impact on neurovascular remodeling and long-term neurological recovery [65]. Intraperitoneal DHA injections and ω-3PUFA dietary supplement significantly reduced tissue atrophy and improved cognition of mice subjected to transient middle cerebral artery occlusion (a model of stroke). Mechanistically, the combined treatment promoted post-stroke neurogenesis and angiogenesis, and reduced glial scar development [77]. From these in vivo studies it comes that combined post-stroke DHA treatment and ω-3 PUFA dietary supplementation may be a potential therapeutic chance for stroke or related brain disorders through reversion of neurovascular damage and cognitive impairment. However, appropriately conducted clinical studies need to be performed to gain insights for human indications.

#### 3.2.5. Biomaterials Biocompatibility/Integration and Wound Healing

Synthetic and biologically derived materials are thoroughly under investigation for various clinical applications to favor wound healing and tissue regeneration (tissues or scaffolds for skin and visceral healing, improvement of bone fracture, bone/cartilage substitution, etc.) as delivery systems for cells, drugs and therapeutics (nanoparticles, hydrogels, liposomes) [65,78,79,80]. To have success the biomaterial should be safe and biocompatible with the biological tissues, in order to avoid rejection and organ failure and for its proper functioning. For certain clinical uses, the induction of angiogenesis is required for integration. Since the beneficial effect of nutraceuticals, biomaterial and nutraceutical sectors may converge to improve biocompatibility and chemical and physical properties of biomaterials. 

A recent study [81] shows the development of a wheat grass bioactive-reinforced collagen-based aerogel system as an instructive scaffold for collagen turnover and angiogenesis. The addition of wheat grass active principles to collagen resulted in a biomaterial with enhanced physicochemical and biomechanical properties. The reinforced biomaterial resulted in a three-dimensional (3D) sponge-like aerogel showing a peculiar structural assembly with increased water retention and substance permeability. This construct design allows nutrient and gas diffusion to facilitate cell adhesion and growth, necessary for the healing of damaged tissues [81]. The angiogenic potential and collagen turnover induced by the engineered aerogel in cell cultures and in in vivo wound healing model make it a potential 3D dressing scaffold useful for tissue healing applications.

Similarly, in the report by Dharunya and coworkers [82] a curcumin cross-linked collagen aerogel was developed with enhanced physico-chemical properties, demonstrating in vitro controlled anti-proteolytic activity and pro-angiogenic efficacy due to enhanced cell adhesion and proliferation.

These limited studies show the possibility to develop biocompatible, biodegradable nutraceutical-reinforced collagen aerogels as instructive scaffolds with antimicrobial properties and proangiogenic activity useful for wound healing applications.

## 4. Pharmacological Issues to Be Solved

A series of critical issues needs however to be considered before the introduction of nutraceuticals in human use for disease therapy. A first issue is related to the pharmaceutical preparation. While natural products seem to be a good option, their concentration, fine structure and bioactivity are very dependent on seasonality, environment and growth conditions. Table 4 contains exemplificative nutraceuticals with effects on angiogenesis and their average concentration in food sources.

In extreme environmental conditions, this can lead to complete loss of activity of the nutraceutical within food staff or extracts. Thus, the consistency of therapeutic preparations should be ensured from multiple batches of products with appropriate quality control or alternatively, by chemical synthesis of the compound of interest. Following preclinical validation of efficacy and discovery of the mechanism of action, specifically designed clinical trials should be run to get information on the best source and formulation of nutraceuticals (food and/or beverages, extracted phytocomplexes, isolated nutraceuticals, synthetic analogues or metabolites). Indeed, as reported in Table 1 clinical trials are running on all these different preparations.

Appropriate formulations should be designed to optimize dosage and preparation of nutraceuticals for the proposed route of administration. Of note, the combination of multiple nutraceuticals as well as the addition to conventional drugs should be assessed, especially taking into account possible pharmacokinetic or pharmacodynamic interactions. According to guidelines, proper toxicological studies are necessary to demonstrate the safety of novel nutraceuticals to be proposed in the market. Once in use, an active pharmaco-vigilance should be conducted by healthcare professionals in order to detect possible adverse reactions, especially when supplementary strategies are given in combination with prescription drugs.

One of the major concerns of nutraceuticals is the extremely low bioavailability due to scarce water solubility and low absorption rate. As a consequence, only negligible levels of the compounds can be found in the circulation and can reach the diseased tissue [83]. Accordingly, there is a great effort to improve nutraceuticals bioavailability, as the use of nano-carriers [99] or hydrophilic carriers [55]. Natural compounds can be packaged into biodegradable polymeric nanoparticles for solid or liquid formulations. Due to the better pharmacokinetic profile of nano-formulations, these preparations demonstrate to retain the activity of the native molecules and to facilitate their targeting to the tissue of interest [100]. For example, EGCG encapsulated in nano-carriers has the same pro-apoptotic and anti-angiogenic effects exerted by the native compound, but with the advantage of a 10-fold lower dose [99], and nano-encapsulated curcumin, kaempferol and berberine have improved in vivo antiangiogenic and anti-tumor effects compared to the free forms [100,101,102]. For curcumin, several oral formulations with hydrophilic carriers are available, and recently, their pharmacokinetic profiles have been investigated in view of use in ocular disorders [55]. However, once designed, the safety and clinical validation of these novel formulations remain to be established [103].

Another concern related to nutraceuticals is the information about the circulating metabolites and their expected biological activities. Indeed, it is not excluded that circulating metabolites can exert the same or opposite biological activities, as seen in one of our previous paper on the dual angiogenic properties of quercetin metabolites [104]. The final result of inhibition or activation of angiogenesis is thus finely dependent on in vivo metabolism by intestinal epithelium, microbiota or liver which control the quality and quantity of circulating metabolites, as well as by the inflammatory/oxidative microenvironment in which the metabolites are co-opted to work. This aspect should be taken into consideration when designing therapeutic or preventive strategies based on food derivatives.

The combination of these two latter aspects of nutraceuticals, nanoformulation and use of active metabolites, has been considered in the study of Bhatt et al. [105] where nanonutraceuticals were designed using the metabolites resulting from the solid phase fermentation of soybean with Bacillus substilis. The efficacy of these novel formulations has been validated for the antioxidant activity and beneficial impact on cognitive defects in an experimental model of Alzheimer’s disease [105].

## 5. Conclusions

A large number of nutraceutical compounds are integral part of the healthy diet and in the past decades a great number of reports supports their beneficial properties in human health, by acting in different signaling pathways of various cell types.

Diet components and selected nutraceuticals have been demonstrated to maintain vascular structure and function or ultimately to induce the formation of capillary-like structures (by inducing endothelial cell growth, migration, and invasiveness). On the other hand, many nutraceuticals mostly derived from plants, are able to inhibit the steps of angiogenesis leading to the inhibition/regression of vascular development. It has to be noted that the same compounds or classes of nutraceuticals, demonstrate both pro- and antiangiogenic properties. This apparent paradox can be explained by considering the context in which the active metabolites work, namely an environment where inflammation and oxidative species are present and concur to disease development together with angiogenesis deregulation. Indeed, most of the nutraceuticals so far studied exert anti-inflammatory and/or anti-oxidative properties.

As discussed earlier [37], the dual activity on angiogenesis by the same nutraceutical seems concentration or dose related: i.e., for red wine polyphenols low doses have been reported to promote angiogenesis, while high doses were antiangiogenic, acting on different cellular and molecular mechanisms [106].

An aspect clearly lacking from the literature about the effect of nutraceutical derivatives on angiogenesis, is vessel normalization. Both in angio-inhibition and in promotion of angiogenesis it is important to obtain physiological or mature vessels, avoiding aberrant neovascularization and highly permeable/dysfunctional vessels. Only one experimental report focused on the effect of nutraceuticals on this feature [107]: the pretreatment with neem leaf glycoprotein resulted in vascular normalization in melanoma and carcinoma bearing mice, giving rise also to immunomodulation. This feature merits to be explored in a more consistent manner.

In conclusion, dietary active components can be developed as promising interventional tools although important aspects remain to be examined, strengthening the knowledge on their concentration-mediated effects, evaluating the effect(s) of specific active molecules and circulating metabolites, designing appropriate formulations and finally establishing their effective usefulness as proangiogenic or antiangiogenic tools to be used alone or in association with drugs.

## Figures and Tables

**Table 1:** List of clinical trial on nutraceuticals and diseases characterized by aberrant angiogenesis (source https://clinicaltrials.gov/).

Identifier	Active Principle (Dose, Route)	Title of the Study (Status)
NCT01521949	Acai Juice 2 ounces by mouth twice daily on a continuous basis	A Phase 2 Study Acai Juice Product in Asymptomatic or Minimally Symptomatic Prostate Cancer Patients With Rising Prostate Specific Antigen (PSA) (Completed, Has results)
NCT03665922	Four tablets (BroccoMax^®^ or placebo) in the morning with breakfast and four tablets in the evening with dinner for daily internal dose of 64 mg of SFN. Four weeks treatment.	Biomarkers of Sulforaphane/Broccoli Sprout Extract in Prostate Cancer (New, Not yet recruiting)
NCT01916239	Two new pomegranate formulations vs. standard pomegranate extract formulation containing 20% punicalagin; 15 days treatment.	Phase I-II Study of Pomegranate Extract Formulations in Colorectal Cancer Patients: Metabolic and Gene Expression Profiling in Tumoral and Normal Colon Tissues (Completed)
NCT02984813	Two pills once daily in the morning for 3 months containing alpha lipoic acid, citicoline, co-enzyme Q10, *Ginkgo biloba* extract, grape seed extract, *N*-acetyl-cysteine, curcumin, and green tea extract or curcumin, bilberry extract, and grape seed extract	Safety and Efficacy of Anti-Oxidants and Anti-inflammatory Agents in Glaucoma and Diabetic Retinopathy (Active, not recruiting)
NCT01646047	Two capsules containing nutritional supplements per day for 6 months (vitamin C, mixed tocopherols/tocotrienols, vitamin D, fish oil, lutein, zeaxanthin, pine bark extract, benfotiamine, green tea extract, curcumin)	Diabetes Visual Function Supplement Study (Completed)
NCT03676309	Nutriceutical Oral Capsule, nutraceutical 920 mg (Bergamot 450 mg, Gymnema 400 mg, Phaseolamine 30 mg, Olea Europaea 10 mg) twice day, 12 weeks	Efficacy and Safety of Nutraceuticals in Patients With Diabetes Mellitus Type II and Dyslipidemia. (Completed)
NCT03593135	15 mL apple cider vinegar (American garden organic vinegar) (containing 5% acetic acid) mixed in 200 mL water during meal at night time (daily, for 3 months)	Effect of Apple Cider Vinegar in Type 2 Diabetics. (Completed)
NCT02969070	LopiGLIK™, Akademy Pharma, 1 capsule/day containing red yeast rice 220 mg (at least 3.3 mg of Monacolin K) + Berberine 531.25 mg + Morus Alba 200 mg (at least 4 mg of Deoxynojirimycin) vs. Armolipid Plus^®^, Meda Pharma, 1 capsule/day containing Berberis aristata d.e. 588 mg (equivalent to Berberine chloride 500 mg) + Red yeast rice 200 mg (equivalent to Monacolin K 3 mg) + Policosanol 10 mg + Folic acid 0.2 mg + Coenzyme Q10 2.0 mg + Astaxanthin 0.5 mg (Daily for 4 weeks)	Effects of Nutraceutical Therapies on Endothelial Function, Platelet Aggregation, and Coronary Flow Reserve (Recruiting)
NCT02772887	Oral l-citrulline, 3 g once per day for 3 weeks	Nutraceutical Citrulline in Pregnancy (Recruiting)
NCT02629952	Three cups of blueberry tea per day for 4 weeks	Metabolic Benefits of Drinking Blueberry Tea in Type 2 Diabetes (Recruiting)
NCT02029833	Regular Canola Oil 60% or 70% oleic acid (daily for 6 weeks)	Canola Oil Multi-Centre Intervention Trial II (COMIT2) (Completed)
NCT01982734	80 mg curcumin were given orally either as native powder, native powder plus phytochemicals, micelles or micelles plus phytochemicals (Pharmacokinetics studies)	Improved Oral Bioavailability of Curcumin Incorporated Into Micelles (completed)
NCT01925287	500 mg curcumin were given orally either as native powder, micronized powder, or liquid micelles (early phase I)	Oral Bioavailability of Curcumin From Micronized Powder and Liquid Micelles in Healthy Young Women and Men (Completed)
NCT01449110	Resveratrol-enriched grape extract (8 mg) (orally, daily for 6 months)	Resveratrol-enriched Grape Extract (Stilvid) in Primary and Secondary Prevention of Cardiovascular Disease (Completed)
NCT01085019	2.8 g/day of cinnamon/oregano/ginger/rosemary/black pepper in capsules during 4 weeks.	Impact of Spices and Herbs on Endothelial Function (Completed)
NCT00296595	2 g/day of fish oil + 500 mL/day of cranberry juice (daily for 12 weeks)	Effects of n-3 Polyunsaturated Fatty Acids and Antioxidants on Postprandial Hyperlipidemia and Vascular Function in Men (Completed)
NCT00654459	Mixture of berberine, policosanol, red yeast, placebo. A tablet one a day for 6 weeks	Effects of Armolipid Plus on Cholesterol Levels and Endothelial Function (Completed)

**Table 2:** Antiangiogenic foods with their main active principles.

Category	Food	Active Principles
Beverages and drinks	Green tea, red wine	Stilbenoids (resveratrol), flavanols (catechins)
Fruits	Strawberries, blackberries, raspberries, blueberries, cranberries, apple, pineapple, cherries, oranges, grapefruit, lemons, red grapes, pomegranate	Carotenoids (lycopene), the most part of flavonoids and in particular glycosides of anthocynidins (anthocyanins), stilbenoids (resveratrol), flavanones (hesperetin),
Vegetables and mushrooms	Soy beans, artichokes, tomatoes, garlic, kale, broccoli, cauliflower, Brussels sprouts, bok choy, lavender, maitake mushrooms, parsley, pumpkin	Flavones (apigenin), isoflavones (genistein), flavonols (quercetin), isothiocyanate (sulfurafane), glycosides of anthocyanidins (anthocyanins)
Oils	Extra-virgin olive oil, grapeseed oil	Oleic acid, phenylethanoids (hydroxytyrosol)
Other	Dark chocolate, ginseng, licorice, turmeric, ginger, nutmeg, cinnamon, red propolis	Glycosides of anthocyanidins (anthocyanins), ginsenoides, phenolic acids (curcumin)
Fish and meat	Tuna, sea cucumber	Omega-3 fatty acids, mucopolysaccharides, saponins

**Table 3:** Foods and related active compounds which prevent endothelial dysfunction.

Categories	Food	Active Principles
Beverages and drinks	Red wine, grape juice, chocolate, green tea, orange juice	Stilbenoids (resveratrol), flavanols (catechins), vitamins
Fruits	Avocados, tomatoes, watermelon, grapefruit, Citrus plants and in general all fruits	Carotenoids (lycopene), glycosides of anthocynidins (anthocyanins), flavanones (hesperetin), vitamins C and E
Vegetables	Leafy greens, soybeans, legumes, red clover, flax, alfalfa, Cruciferae family, onions, shallots, garlic	Flavones (luteolin, apigenin), isoflavones (genistein), flavanols (quercetin), isothiocyanate (sulfurafane), glycosides of anthocyanidins (anthocyanins), vitamins C and E, folate, l-arginine (from plant proteins)
Oils	Olive oil, flaxseed oil, canola oil, soybean oil, cod liver oil, herring oil, salmon oil	Oleic acid, phenylethanoids (oleuropein, hydroxytyrosol, tyrosol), omega-3 fatty acids
Other	Nuts, cereals, grains,	Vitamin E
Fish	Anchovy, bass, bluefish, capelin, dogfish, eel, herring, mackerel, mullet, rockfish, sablefish, salmon, saury, scad, smelt, sturgeon, trout, tuna, whitefish	Omega-3 fatty acids

**Table 4:** Exemplificative nutraceuticals able to affect angiogenesis and endothelial functions and their average concentration in food sources.

Chemical Formula	Nutraceutical [Food Source]	Average Concentration	Reference for Biological Activity
**Antiangiogenic nutraceuticals**			
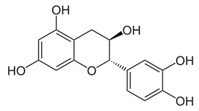	Catechins (flavanol) [green tea, chocolate]	30–250 mg/kg fresh weight 60–800 mg/L infusion [83,84]	[85,86,87]
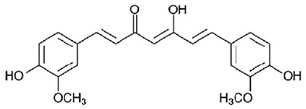	Curcumin [Curcuma longa]	3.14 g/100 g of turmeric powder [83,88]	[89,90]
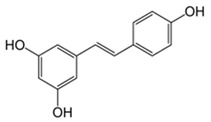	Resveratrol [grapes, berries, peanuts, etc.]	0.3–7 mg aglycones/L and 15 mg glycosides/L in red wine [83,84]	[91,92]
**Proangiogenic/endothelial protective nutraceuticals**			
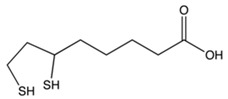	Alpha lipoic acid [kidney, heart, liver, spinach, broccoli, and yeast extract] [93]	0.1–2.6 mg/kg dry weight	[94]
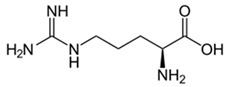	l-arginine [turkey breast, pork loin, chicken, pumpkin seed, soybean, peanuts, spirulina, dairy, chickpeas, lentils] [95]	0.1–3.13 g/100 g of plant food 0.08–1.74 g/100 g of animal food	[96]
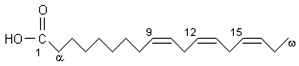 Alpha-linolenic acid	ω3-PUFA [fish oil, flaxseed oil, canola oil, soybean oil, olive oil] [97,98]	1–5 mg/100 g of fish 7 mg/tablespoon of flaxseed oil 7.9 g/100 g olive oil	[75,76,77]
